# Innovative method to grow the probiotic *Lactobacillus reuteri* in the omega3-rich microalga *Isochrysis galbana*

**DOI:** 10.1038/s41598-022-07227-y

**Published:** 2022-02-24

**Authors:** Eleonora Colantoni, Francesca Palone, Vincenzo Cesi, Beatrice Leter, Giulia Sugoni, Ilaria Laudadio, Anna Negroni, Roberta Vitali, Laura Stronati

**Affiliations:** 1 Department of Molecular Medicine, Sapienza University of Rome, Viale Regina Elena 291, 00161 Rome, Italy; 2 Division of Health Protection Technologies, ENEA, Via Anguillarese 301, 00123 Rome, Italy; 3 Division of Protection and Enhancement of the Natural Capital, ENEA, Via Anguillarese 301, 00123 Rome, Italy

**Keywords:** Biotechnology, Microbiology, Health care

## Abstract

Microalgae are natural sources of valuable bioactive compounds, such as polyunsaturated fatty acids (PUFAs), that show antioxidant, anti-inflammatory, anticancer and antimicrobial activities. The marine microalga *Isochrysis galbana* (*I. galbana*) is extremely rich in ω3 PUFAs, mainly eicosapentaenoic acid (EPA) and docosahexaenoic acid (DHA). Probiotics are currently suggested as adjuvant therapy in the management of diseases associated with gut dysbiosis. The *Lactobacillus reuteri* (*L. reuteri*), one of the most widely used probiotics, has been shown to produce multiple beneficial effects on host health. The present study aimed to present an innovative method for growing the probiotic *L. reuteri* in the raw seaweed extracts from *I. galbana* as an alternative to the conventional medium, under conditions of oxygen deprivation (anaerobiosis). As a result, the microalga *I. galbana* was shown for the first time to be an excellent culture medium for growing *L. reuteri*. Furthermore, the gas-chromatography mass-spectrometry analysis showed that the microalga-derived ω3 PUFAs were still available after the fermentation by *L. reuteri.* Accordingly, the fermented compound (FC), obtained from the growth of *L. reuteri* in *I. galbana* in anaerobiosis, was able to significantly reduce the adhesiveness and invasiveness of the harmful adherent-invasive *Escherichia coli* to intestinal epithelial cells, due to a cooperative effect between *L. reuteri* and microalgae-released ω3 PUFAs. These findings open new perspectives in the use of unicellular microalgae as growth medium for probiotics and in the production of biofunctional compounds.

## Introduction

Nutritious and sustainable foods with a low impact on the environment, economy and society represent today a global challenge. Microalgae, microscopic photosynthetic organism, have gained a lot of interest over the years as they have a wide range of applications including the development of biofuels and biofertlizers^1,2^. Furthermore, microalgae are natural sources of valuable bioactive compounds showing antioxidant, anti-inflammatory, anticancer and antimicrobial activities, such as vitamins, essential amino acids, polyunsaturated fatty acids (PUFAs), minerals, carotenoids and enzymes^3–7^.

In particular, microalgal lipids comprising of ω3 PUFAs, mainly eicosapentaenoic acid (EPA, 20:5 ω3) and docosahexaenoic acid (DHA, 22:6 ω3), give microalgae a high added value for their effectiveness in the treatment of several disorders, such as cardiovascular syndromes, diabetic disease, Alzheimer’s disease, growth and brain development of infants and cancer^8,9^. Very recently, a role of ω3 PUFAs in impairing detrimental gut bacteria, such those producing trimethylamin, has also been suggested^10^.

The marine microalga *Isochrysis galbana* (*I. galbana*), extremely rich in EPA and DHA, is a valuable source for human and animal nutrition and represents a potentially promising therapeutic tool for the management of several diseases^11–16^.

Probiotics, viable non-pathogenic microorganisms providing health benefits to the host, are suggested as adjuvant therapy against diseases associated with gut dysbiosis^17–20^. *Lactobacillus reuteri* (*L. reuteri*), a commensal-derived anaerobic probiotic that resides in the human gastrointestinal tract, is one of the most widely used probiotics showing multiple beneficial effects on host health^21–24^. Recent evidence highlights the role of *L. reuteri* in controlling the growth and survival of pathobionts correlated with infectious or chronic gastrointestinal diseases, such as the adherent-invasive *Escherichia coli* (AIEC)^25,26^.

This study aimed to propose an innovative method to promote the growth of the probiotic *L. reuteri* in the raw seaweed extracts from *I. galbana* as an alternative to the conventional medium, under conditions of oxygen deprivation (anaerobiosis). Main advantages of this method were the low-cost and the possibility of collecting the fermented medium at the end of the growth, administering it together with the probiotic, eluding the purification step. The microalga *I. galbana* was shown for the first time to be an excellent culture medium for the growth of *L. reuteri*. Moreover, the ω3 lipids present in the seaweed were shown to be still available after the fermentation process. The fermented compound (FC), obtained from the growth of *L. reuteri* in *I. galbana* in anaerobiosis, was able to significantly reduce the AIEC adhesiveness and invasiveness to intestinal epithelial cells, due to a cooperative effect between *L. reuteri* and microalga-released ω3 PUFAs.

## Materials and methods

### Microalgal and bacterial strains

Dried powder of *I. galbana,* (freeze dried biomass for aquaculture, batch number ISO15SPRI2, Archimede Ricerche srl, Camporosso, Italy) was sterilized by UV under hood, weighed in sterile conditions, solubilized with phosphate buffered saline (PBS) and left on the rocker for 30 min*.*

The adherent-invasive AIEC strain LF82 (kindly provided by Prof. Arlette Darfeuille-Michaud, Université Clermont-Auvergne, Clermont-Ferrand, France) was cultured in Tryptone Soy Agar (TSA; plates Oxoid, Basingstoke, UK) for 24 h at 37 °C and then sub-cultured in Tryptone Soy Broth (TSB; Oxoid, Basingstoke, UK) with overnight incubation at 150 rpm, 37 °C.

Powder of *L. reuteri* DSM17398 (BioGaia, Stockholm, Sweden) was kept at − 20 °C, inoculated in commercial medium De Man, Rogosa and Sharpe (MRS; Sigma-Aldrich, St. Louis, USA) and incubated overnight, 37 °C without agitation.

### Cell culture

Human colorectal adenocarcinoma cell line, CACO2, was obtained from the American Type Culture Collection (ATCC, Rockville, MA, USA). Cells were grown at confluence at 37 °C in Dulbecco’s minimum essential medium (DMEM; Gibco, Life Technologies, Carlsbad, CA, USA), supplemented with 10% inactivated fetal bovine serum (FBS; Euroclone, Milan, Italy) and 2 mM l-glutamine, 100 U/ml penicillin and 100 g/ml streptomycin (Biochrom, Berlin, Germany).

### Anaerobic growth of *L. reuteri*

In order to ensure no air/oxygen contact during fermentation, 2 × 10^6^ CFU/ml of *L. reuteri* were inoculated in 10 ml of MRS or I. galbana solubilized in PBS (36 mg/ml). The solutions were aliquoted in 5 vials (2 mL each), fill up to the edge, then closed and sealed with parafilm. Vials were incubated anaerobically without agitation at 37 °C for 120 h (5 days) and opened only the day of the experiment.

The bacterial growth was evaluated at different times (24, 48, 72, 96, 120 h) by plating serially diluted samples in PBS on MRS agar plates (1.2% agarose) and incubated at 37 °C for 24 h. Resulting colonies were counted and the viability (CFU/ml) value was calculated based on the plated dilution.

### Lipid extraction

Lipids were extracted from *I. galbana* (36 mg/ml) solubilized in PBS and FC of a single experiment and the analysis was performed in duplicate.

Samples were freeze-dried for 2 days at − 40 °C and 60 mBar pressure by freeze-dryer (Edwards). Each sample (5 mg) was resuspended with 1 ml of dichloromethane (DCM) and 0.5 ml of methanol/sulfuric acid (MeOH/ H_2_SO_4_) and sonicated for 1 h at 50 °C, 40 kHz frequency. Hexane (1 ml) was used as extracting solvent and, after agitation, calcium carbonate (16 mg) and H_2_O (1 ml) were added and samples were centrifugated for 5 min at 2000 rpm. The separation of polar from apolar phase was repeated twice and finally the latter was dried with nitrogen flow (4 ml for each sample).

### Gas-chromatography mass-spectrometry (GC–MS)

GC–MS analysis was performed by a 7890A gas chromatograph (Agilent) with capillary columns SBP-2331 (Sigma-Aldrich) [60 m, 0.25 mm inner diameter (ID), 0.2 µm film thickness]. Helium was used as carrier gas at a linear velocity of 36.26 cm/s and 1 µl of each sample was injected splitless. The initial column temperature was 40° and held 4 min, ramped to 140° at the rate of 20°/min, ramped to 220° at the rate of 2°/min and held 1 min and then finally increased to 260° at the rate of 10°/min and kept at this temperature for 5 min. The mass spectra were recorded using a 5975C mass spectrometer (Agilent) in full scan mode from 45 to 450 m/z and 240°. The fatty acids concentration of each sample was determined using the software Xcalibur (Thermo Scientific, Waltham, USA) and 37 Component FAME Mix (Supelco, USA) was used as external standard for calibration.

### Co-culture of AIEC LF82 and *L. reuteri* in *I. galbana*

To test the ability of AIEC LF82 and *L. reuteri* to grow in *I. galbana*, 1.3 × 10^6^ CFU/ml of *L. reuteri* and 1.4 × 10^7^ CFU/ml of LF82 were inoculated in 10 ml of *I. galbana* solubilized in PBS (36 mg/ml), aliquoted in 5 vials, capped and incubated anaerobically without agitation at 37 °C for 120 h (5 days).

The *L. reuteri* and LF82 growth was evaluated at different times (24, 48, 72, 96, 120 h) by plating serially diluted samples in PBS respectively on MRS agar plates (1.2% agarose) and TSA and incubated at 37 °C for 24 h. Resulting colonies were counted and the viability (CFU/mL) value was calculated based on the plated dilution.

### AIEC adhesion and invasion assay

#### Adhesion assay

CACO2 cells were grown on 24-well plates at confluence (3 × 10^5^ cells) and infected with LF82 (3 × 10^6^ CFU), or LF82 + *L. reuteri* (3.5 × 10^6^ CFU), or LF82 + *I. galbana* (100 µl), or LF82 + FC (100 µl) at 37 °C for 3 h. The final volume was 1 ml/well and 100 µl of *I. galban*a and FC were taken before and after fermentation without any further concentration step. To quantify the adherence of LF82, we followed the protocol of Darfeuille-Michaud et al.^27^. Briefly, infected cells were washed twice in PBS and lysed for 10 min with 0.5 ml of 0.1% Triton X-100 in PBS buffer. Adherent bacteria were recovered and plated on TSA plates. The latter were incubated at 37 °C overnight and then the colonies were counted for statistical analysis.

#### Invasion assay

CACO2 cells were infected and incubated as above. For invasion assay, we followed the protocol of A. Darfeuille-Michaud et al.^32^. Briefly, after incubation, cells were washed twice in sterile PBS and then incubated in DMEM and McCoy’s medium, respectively with 0.1 mg/ml gentamicin for 1 h to kill the extracellular bacteria. Cells were washed twice in sterile PBS. Lysis, incubation and counts were performed as in the adhesion assay. To ensure maximum reproducibility, accuracy and statistical significance, adhesion and invasion assays were carried out simultaneously in triplicates. To obtain an accurate count of adhesive bacteria, the number of invasive colonies was subtracted from the number of the adhesive ones.

### Statistics

Data are given as mean ± standard deviation. All experiments were repeated three times. Comparison between groups was performed by a two-tailed Student t-test (significance taken as *P* < 0.05)*.*

## Results

### The unicellular microalga *I. galbana *was shown to be an adequate culture medium for the growth of the probiotic *L. reuteri*

**Figure 1:**
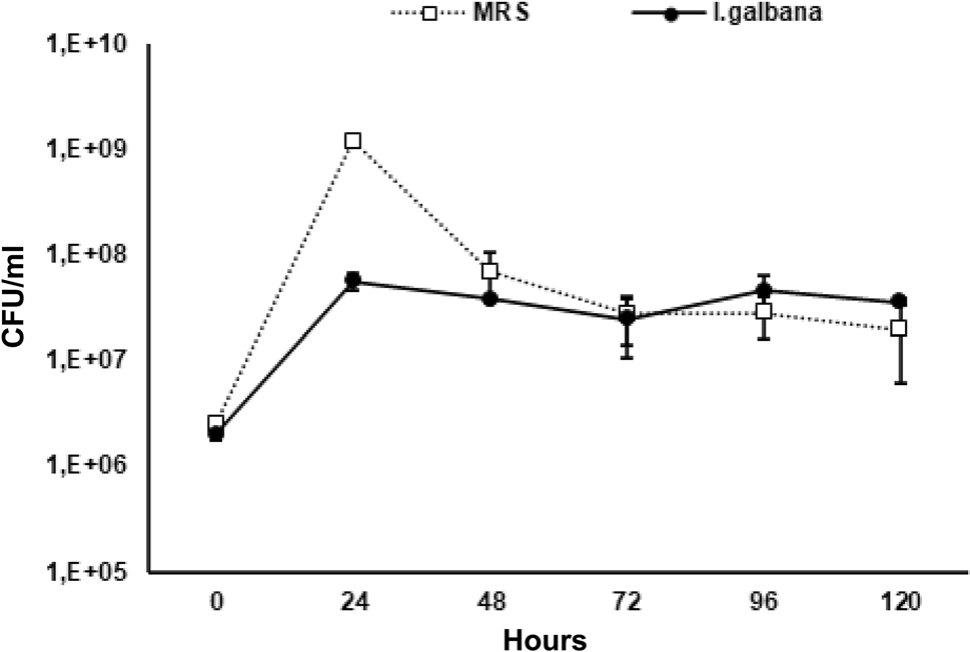
Anaerobiotic growth of *L. reuteri* in the marine microalga *I. galbana*. The probiotic *L. reuteri* was able to grow in *I. galbana* as well as in the commercial medium. *L. reuteri Lactobacillus reuteri*, *I. galbana Isochrysis galbana*, *MRS* commercial medium.

The probiotic *L. reuteri* was inoculated at a concentration of 2 × 10^6^ CFU/ml in physiological solution containing *I. galbana* (36 mg/ml) or commercial medium (MRS) and placed at 37 °C in anaerobiosis. The growth was followed for 5 days. Results showed that, although in the first 24 h, the growth of *L. reuteri* is highest in the conventional medium than *I. galbana*, however, at the end of the 5 days of fermentation the growths are comparable, reaching the final concentration of 3.5 × 10^7^ and 1.9 × 10^7^, respectively (Fig. 1).

### The microalga-derived ω3 PUFAs were still available after the fermentation by *L. reuteri*

**Figure 2:**
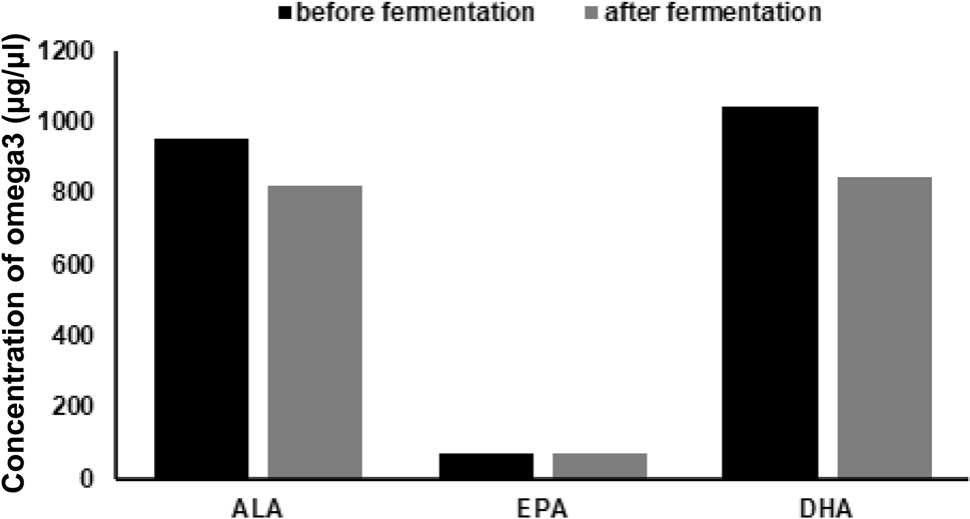
Microalga omega3 profile before and after *L. reuteri* fermentation. Lipidomic analysis by GC–MS of *I. galbana* showing PUFA-omega3 availability. *L. reuteri Lactobacillus reuteri*, *GC–MS* gas-chromatography mass-spectrometry, *I. galbana Isochrysis galbana*, *PUFA* polyunsaturated fatty acids, *ALA* alpha-linoleic acid, *EPA* eicosapentaenoic acid, *DHA* docosahexaenoic acid.

The GC–MS lipidomic analysis of *I. galbana* confirmed that the microalga was rich in ω3 PUFAs, especially in DHA. More interestingly, the analysis showed that the availability of DHA and EPA was similar before and after fermentation by *L. reuteri*. Indeed, the amount of EPA was unchanged, while alpha-linoleic acid (ALA) and DHA underwent a small variation, between 15 and 20% less after fermentation (Fig. 2). Therefore, the FC was rich in probiotic as well as ω3 PUFAs.

### The unicellular microalga *I.* galbana promoted the growth of the probiotic* L. reuteri* compared to the pathobiont AIEC LF82

The harmful AIEC LF82 was co-cultured with the *L. reuteri* in *I. galbana* to investigate the ability of LF82 to compete with the probiotic in the microalga culture medium.

**Figure 3:**
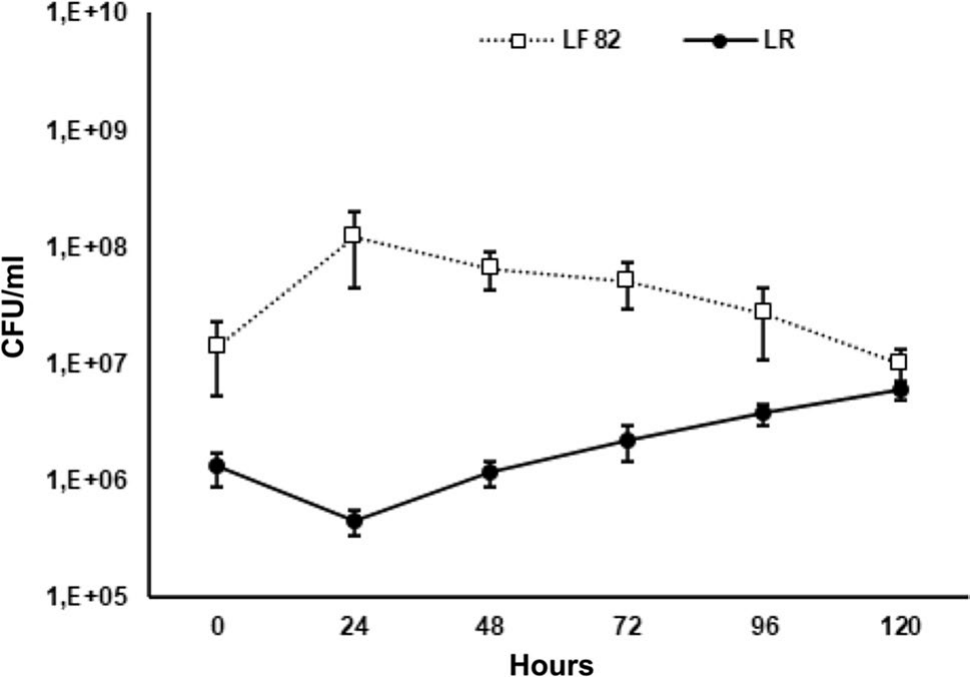
Competitive growth of *L. reuteri* and AIEC LF82 in *I. galbana*. Differential growth of LF82 (open square) and *L. reuteri* (filled circle) in *I. galbana. AIEC* adherent-invasive *Escherichia coli*, *L. reuteri Lactobacillus reuteri*, *I. galbana Isochrysis galbana.*

Interestingly, although LF82 had been inoculated at a concentration of 1 log higher, however, the growth curve of LF82 decreased after the first 24 h, while that of *L. reuteri* was improved, reaching quite the same concentration (9.9 × 10^6^ and 6 × 10^6^, respectively) after five days (Fig. 3), suggesting that *I. galbana* promoted the growth of *L. reuteri* as compared to LF82.

### The FC derived from the 5 days-growth of *L. reuteri* in *I. galbana* strongly limited the adhesiveness and invasiveness of LF82 to intestinal epithelial cells

The human epithelial colorectal adenocarcinoma cells, CACO2, are a recognized in vitro model of intestinal epithelial barrier. Hence, confluent CACO2 cells were used to assess the ability of the FC to control the adhesiveness and invasiveness of AIEC LF82, better that the probiotic alone. Confluent CACO2 were exposed for 3 h to LF82 alone (3 × 10^6^ CFU) or LF82 + *L. reuteri* (3.5 × 10^6^ CFU) or LF82 + *I. galbana* (100 µl) or LF82 + FC (100 µl).

**Figure 4:**
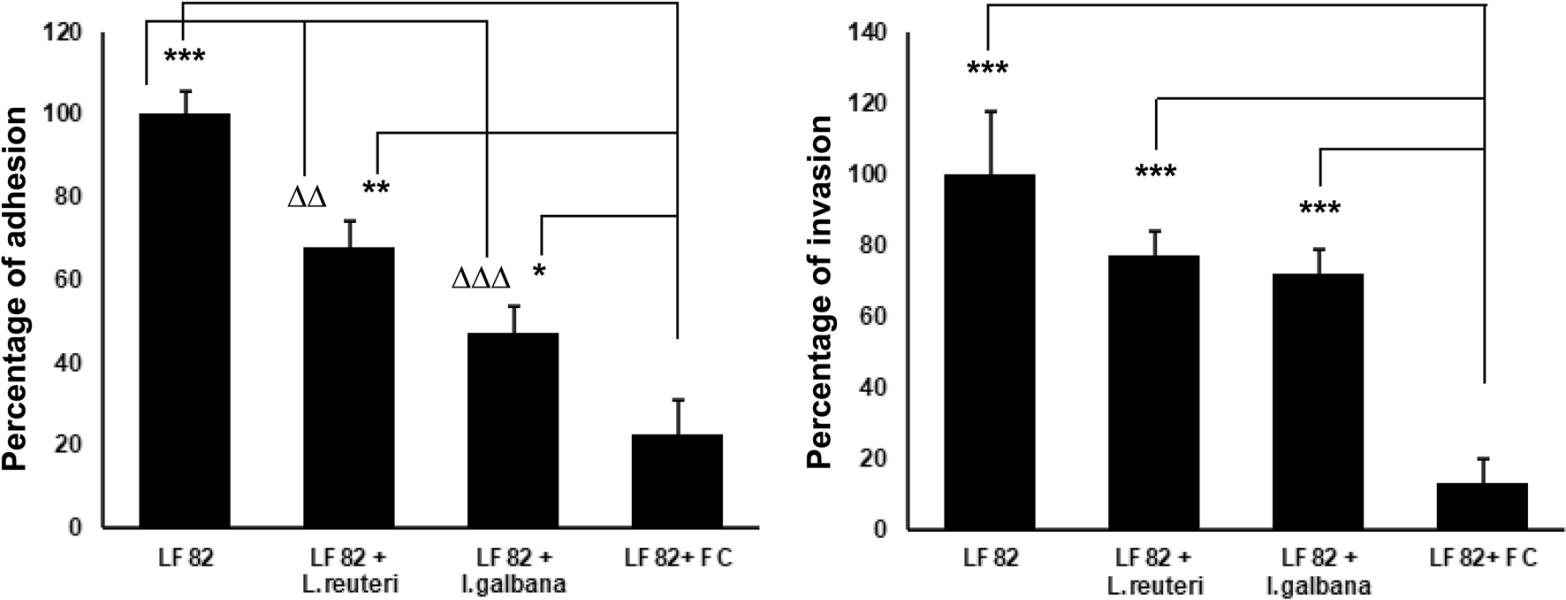
FC reduced the adhesiveness and invasiveness of AIEC LF82 to CACO2 cells. FC significantly reduced the adhesion and invasion of AIEC LF82 to CACO2 cells compared to the probiotic *L. reuteri* and microalga *I. galbana* alone. *AIEC* adherent invasive *Escherichia coli*, *CACO2* human colorectal adenocarcinoma cell line, *FC* fermented compound, *L. reuteri Lactobacillus reuteri*, *I. galbana Isochrysis galbana* */^∆^P < 0.05; **/^∆∆^P < 0.01; ***/^∆∆∆^P < 0.001.

Results confirmed that *L. reuteri* was able to reduce the pathogenicity of LF82. Surprisingly, the microalga *I. galbana* alone was able to decrease the AIEC harmfulness, as well. The FC significantly reduced the adhesion (P = 0.002) and invasion (P = 0.0002) of LF82 compared to the probiotic or the microalga administered individually (Fig. 4).

## Discussion

To date, probiotic production has almost exclusively been carried out using conventional batch fermentation and suspended cultures, but there is an emerging interest from the scientific community and increasing demand from the business world to explore and set up innovative fermentation technologies.

Here, a very innovative method promoting the growth of the probiotic *L. reuteri* in the microalga *I. galbana* under anaerobiosis condition has been proposed. Advantages of this protocol are several. First, the cost is low since the marine microalga is used as a raw material for fermentation; further, the probiotic does not need to be purified at the end of fermentation but can be administered together with the culture medium which still contains the ω3 lipids that are beneficial for the host organism; finally, since probiotics must colonize an oxygen-deprived gut environment, the fermentation of the microalga in anaerobiosis can be thought as a form of pre-adaptation of probiotics, improving their survival in the bowel.

Recently, marine microalgae have been recognized as an efficient way to derive high-value products with biomedical and nutritional applications. However, in our study a unicellular microalga has been used as a growth medium for probiotics for the first time. Remarkably, our results showed that the probiotic *L. reuteri* was able to grow in *I. galbana* as well as in the conventional culture medium. Further, the ω3 lipids, in particular EPA, were still available in the medium after fermentation. Current evidence shows that ω3 lipids may regulate the antioxidant signaling pathway and modulate inflammatory processes suggesting a pivotal role in clinical therapy^28^. Therefore, the microalgae-probiotic combination showed great potential for generating a novel functional product, here called the fermented compound (FC).

The advantage of combining microalgae and probiotics has already been highlighted, albeit with different purposes: indeed, a recent paper showed that adding the microalgae *Chorella vulgaris* to the *Lactobacillus* spp. growth medium accelerated the growth and the metabolic activity of the bacterium, suggesting that this combination allowed for the development of innovative, functional products with advantageous characteristics of the final product^29^.

The FC properties were investigated through a challenge against AIEC bacteria harmfulness. The AIEC uniquely benefit from host genetic alterations or specific environments to promote their adhesion to intestinal mucosa with an inflammatory response^30^. Remarkably, prevalence of AIEC bacteria in the gut mucosa can involve up to 60% of patients with IBD^31^: thus, AIEC-targeting strategies, limiting their mucosa colonization, could represent a therapeutic option in managing patients with intestinal inflammation. On this point, the ability of *L. reuteri* to reduce the pathogenicity of enteroinvasive *Escherichia coli*^32,33^, including AIEC^26^ has been already proven.

Intriguingly*, I. galbana* was shown to be an excellent growth medium for the probiotic *L. reuteri* but not for harmful species such as LF82: indeed, co-culturing LF82 and *L. reuteri*, after the first 24 h, the growth of LF82 decelerated while *L. reuteri* steadily grew until the end of a five-day period. These results suggested that *I. galbana* may counteract the AIEC growth while favoring *L. reuteri* development*.*

It was of interest that the treatment with FC prevented the AIEC adhesiveness and invasiveness to epithelial cells more effectively than the probiotic alone, using confluent CACO2 cells as a model of gut barrier. This effect likely resulted from a synergism between *L. reuteri* and the microalga-released ω3 PUFAs.

It is worth noting that it is the first time that *I. galbana* is shown to significantly decrease the adhesion and invasion of LF82 to CACO2 cells with an efficiency comparable to that of *L. reuteri*. Although several microalgae, including *I. galbana*, have previously been suggested as forthcoming candidates to inhibit the growth of gram-positive bacteria^34^, however, to our knowledge, their potential in controlling pathobionts has not yet reported.

## Conclusions

Current evidence indicates that microalgae have the capability to become a novel source of bioactive molecules, especially with a view to enhance the nutritional and functional quality of foods. The novelty of the present study was to provide evidence that unicellular microalgae may also represent a reliable culture medium for growing the probiotics. The microalga *I. galbana*, that we used as a model, is an adequate culture medium for the probiotic *L. reuteri* with the resulting fermented compound showing beneficial effects in limiting the AIEC adhesiveness and invasiveness to intestinal epithelial cells, likely due to its richness in DHA and EPA lipids.

Intriguingly, the *L. reuteri* grown in *I. galbana* should be considered a true novel vegetarian probiotic since free from all animal-derived ingredients differently from probiotics grown in the traditional culture medium.

## Data Availability

The data that support the findings of this study are available from the corresponding author [L. S.], upon reasonable request.
